# Development of a Novel Biobased Polyurethane Resin System for Structural Composites

**DOI:** 10.3390/polym14214553

**Published:** 2022-10-27

**Authors:** Oihane Echeverria-Altuna, Olatz Ollo, Izaskun Larraza, Cristina Elizetxea, Isabel Harismendy, Arantxa Eceiza

**Affiliations:** 1 TECNALIA, Basque Research and Technology Alliance, Science and Technology Park, Gipuzkoa Mikeletegi Pasealekua 2, 2009 Donostia-San Sebastian, Spain; 2 ‘Materials + Technologies’ Research Group (GMT), Department of Chemical and Environmental Engineering, Faculty of Engineering of Gipuzkoa, University of the Basque Country, Plaza Europa 1, 20018 Donostia-San Sebastian, Spain

**Keywords:** biobased composite, structural application, polyurethane, RTM, thermosetting resin

## Abstract

Polyurethanes are gaining increasing interest for their use as structural components subjected to cyclic loads, such as leaf springs. Thermoset polyurethane (PUR) based technology offers some advantages, such as fatigue resistance, low viscosity, and fast curing. However, current PUR formulations present two major drawbacks: their petrochemical origin and high reactivity. The aim of this work was to develop a novel biobased PUR (BIO-PUR) with the required mechanical properties and processability for manufacturing structural composites by resin transfer moulding (RTM). For this purpose, a high functionality and high hydroxyl index castor-oil-based polyol was used combined with a biobased glycerol (BIO-Gly) to increase the crosslinking density and improve the final properties of the BIO-PUR. The viscosity and reactivity of the different systems were studied by means of rheology tests and differential scanning calorimetry (DSC). Thermal and mechanical properties were studied by dynamic mechanical analysis (DMA) and flexural tests. Furthermore, the RTM process of a representative part was simulated and validated through the manufacturing and testing of plates. The properties of the BIO-PUR resin systems were strongly influenced by the addition of biobased glycerol and its effect on the crosslinking density. The combination of a high functionality and hydroxyl index biobased polyol with the biobased glycerol resulted in a high-performance BIO-PUR with the required reactivity and final properties for structural applications.

## 1. Introduction

Consumer demand for low environmental impact solutions and associated policies and legislations are pushing the composites industry to seek more sustainable solutions [1]. Polyurethane resins (PURs) represent an interesting alternative to the commonly used epoxy resins for structural composites, thanks to their higher toughness and fatigue resistance that allow to further extend the composites’ service life [2,3,4,5]. This is the reason why, in recent years, the use of polyurethane thermosets as a matrix for high-performance composites in the automotive and wind energy sectors is being explored [6,7].

However, the commercial PUR systems present some environmental drawbacks, such as their petrochemical origin [7,8,9,10,11,12]. For this reason, biobased polyurethanes (BIO-PURs) based on polyols derived from vegetable oils are being developed for a huge variety of applications [13]: biomedical applications [9,14,15,16], insulation [17,18,19,20], coatings [21,22,23,24], and adhesives [12,25,26], among others. The most used oils are castor, soybean, sunflower, palm, canola and eucalyptus oils [27,28,29,30,31,32,33,34,35,36]. However, despite the intensive investigation on BIO-PURs, there is not yet a high-performance formulation suitable for structural applications such as automotive parts [37,38,39].

The BIO-PUR characteristics are directly correlated with the polyol’s nature, so a good selection of the biobased polyol is critical. In a previous work, it was observed that, using a high functionality and high hydroxyl index castor-oil-based polyol, it was possible to synthesise BIO-PURs that could be suitable for structural applications. The reactivity, viscosity profile, and most of the final properties were equivalent to a reference petrochemical-origin high-performance PUR for resin transfer moulding (RTM) [40]. However, some of the properties, such as the elastic modulus, were lower. Nevertheless, these properties could be enhanced by the addition of other components on the formulation, such as crosslinking agents to increase the network rigidity [41,42].

The aim of this work was to develop a novel BIO-PUR formulation with the required reactivity and properties for the RTM manufacturing of structural composites. More specifically, the target application was an automotive leaf spring.

In order to achieve the desired rigidity, besides a castor-oil-based polyol, a biobased glycerol, BIO-Gly, was also used in the synthesis of the BIO-PUR. Glycerol has proven to be efficient for increasing the crosslinking density, T_g_, and toughness in non-structural BIO-PURs due to its low molecular weight [13,35,43]. Moreover, glycerol can be produced from renewable sources as a byproduct of transesterification reactions in biodiesel plants or of saponification and hydrolysis reactions in oleochemical plants [44].

Another aspect that has to be considered is the BIO-PUR reactivity. A biobased alternative suitable for structural composites should have low initial viscosity, latency, and fast curing to allow fast and low-cost manufacturing processes such as RTM [45]. For this purpose, a previously developed delayed action catalyst based on epoxide and LiCl [45] was added to the BIO-PUR formulation.

The viscosity and reactivity of the different BIO-PUR resin systems were studied by means of rheology tests and differential scanning calorimetry (DSC). Furthermore, to evaluate the effect of BIO-Gly on the final properties, dynamic mechanical analysis (DMA) and flexural tests were carried out.

In order to evaluate the different alternatives and find the best process parameters of the RTM manufacturing, a representative automotive composite part was simulated with ESI’s PAM-RTM software. Finally, once the process parameters were optimised, a composite part was manufactured and characterised. Results showed the suitability of the developed BIO-PUR formulation for structural automotive applications.

## 2. Materials and Methods

### 2.1. Materials

In this work a commercial polymeric methylene diphenyl diisocyanate (pMDI) (Voraforce TR 1500-Isocyanate, isocyanate (NCO) equivalent weight = 136 g eq^−1^, and viscosity = 130 mPa s) supplied by Dow Chemical (Milan, Italy) was employed. The NCO content was determined according to ASTM D2572-97.

In the case of polyols, two different components were used. The first polyol, used as a reference, was a petrochemical-origin polyether-polyol, supplied by Dow Chemical (Voraforce TR 1551-Polyol, OH-index = 527 mg KOH g^−1^, and viscosity = 750 mPa s, Milan, Italy). The second polyol derived from castor oil was supplied by Vertellus (Polycin T-400, functionality = 3, OH-index = 400 mg KOH g^−1^, and viscosity = 1500 mPa s). The hydroxyl indexes of Voraforce 1551 and Polycin T-400 were determined according to ASTM D 4274-88.

A biobased glycerol, BIO-Gly, supplied by Sigma Aldrich, was used as a low-molecular-weight crosslinking agent.

The catalyst employed was a two-component system formed by an epoxide (1,4-butanediol diglycidyl ether, BDDE) and a halide salt (LiCl) dissolved in a low-molecular-mass biobased cycloaliphatic diol (1,4:3,6-dianhydro-D-glucitol or D-isosorbide, DAS). All the catalyst components were supplied by Sigma Aldrich, St. Louis, MO, USA.

A unidirectional glass fibre specifically developed for components subjected to cyclic loadings (Ultra Fatigue UD, weaving pattern: E-glass unidirectional non-crimp fabric (NCF), areal weight: 1176 ± 64 g m^−2^), supplied by Saertex (Saerbeck, Germany), was selected as reinforcement.

Four PUR systems were synthesised, three of them biobased and one of them petroleum-based, as reference. The isocyanate index was maintained constant (equal to 1.2) for all the PUR systems studied. Designation and composition of the PUR systems are summarised in Table 1. All formulations are based on 100 parts by weight of polyol (pbw).

In the case of the glycerol-containing mixtures (BIO-PUR2 and BIO-PUR3), the BIO-Gly was previously mixed with the biobased polyol. The components were prepared as shown in Figure S1 (from the Supplementary Materials).

The addition of the catalysts (PUR-REF and BIO-PUR3) was performed following the protocol described in a previous work [45].

Both only the matrix (neat resin) and the fibre reinforced matrix (composite) plates were prepared for testing. The neat plates were manufactured by casting the resin into a mould and curing it in an oven at 120 °C for 1 h. Composite plates with 47% of fibre volume content of high-fatigue-resistance (ultra-fatigue) unidirectional glass fibre were prepared with the resin transfer moulding (RTM) process in a rectangular mould with a transparent glass cover. RTM injection test was performed at 60 °C and at pressure gradients ranging from 0.5 bar to 3 bar in order to maintain a filling rate between 25 and 100 mL min^−1^. Then, the samples were post-cured for 1 h at 120 °C.

### 2.2. Methods

#### 2.2.1. Rheological Characterisation

Rheological tests were carried out on a HAAKE RheoStress 6000 Rheometer (Thermo Fisher Scientific, Waltham, MA, USA), running in an oscillating stress mode at a frequency of 1 Hz. The amplitude was held constant in the linear viscoelastic range (LVR) throughout the test. A gap separation of 1 mm and disposable parallel plates of 60 mm diameter were used. Experiments were performed at dynamic or temperature sweep test conditions. Temperature sweep tests were performed from 25 to 200 °C at a constant heating rate of 5 °C min^−1^. Storage and loss moduli (G’ and G’) and complex viscosity (η*) were measured over temperature.

#### 2.2.2. Differential Scanning Calorimetry (DSC)

DSC tests were carried out on a TA Instruments DSC Q100 (TA Instruments, New Castle, DE, USA) calorimeter in both dynamic and isothermal conditions. The dynamic experiments were performed from 20 to 200 °C at 10 °C min^−1^. Isothermal experiments were performed at temperatures ranging from 80 to 120 °C. All samples were subjected to a subsequent dynamic scan from 20 to 200 °C at 10 °C min^−1^ to determine the residual heat of reaction. The total heat of reaction (HT) was calculated from the integration of the area of the exothermic peaks.

The curing rates (*dα*/*dt*) obtained from the heat flow curves of the dynamic and isothermal DSC scans (Equation (1)) were integrated to calculate the degree of cure (*α*) profiles (Equation (2)).

(1) $$H=\frac{dH}{dt}=\frac{d\alpha }{dt}\;H_{T}$$

(2) $$\alpha =\int_{0}^{t}\frac{d\alpha }{dt}\;dt$$

where *H* is the instantaneous heat that evolved during the polymerisation reaction of the resin, and *H_T_* is the total heat of the curing process.

#### 2.2.3. RTM Process Simulation

PAM-RTM, the resin moulding module within ESI’S PAMCOMPOSITES composites manufacturing software, was used to simulate the RTM process. Isothermal simulations with a linear lateral injection strategy boundary condition were performed at 120 and 60 °C at constant injection pressures or constant flow rates.

#### 2.2.4. Dynamic Mechanical Analysis (DMA)

DMA tests were carried out using the Gabo Eplexor100N (Netzch, Selb, Germany) dynamic mechanical analyser. Temperature scans were performed from −40 to 200 °C at 2 °C min^−1^ heating rate and at a frequency of 1 Hz. The sample dimensions were 2.2 × 5 × 50 mm^3^. Tests were performed in flexural mode. The T_g_ of the PUR resin systems was taken at the temperature value of the maximum of tan δ, *Tα* [42,46].

#### 2.2.5. Mechanical Properties

The flexural tests were carried out at room temperature using the Instron 5967 (Instron, Norwood, MA, USA) equipment, with a 3-point bending device, according to the ISO 178 standard for resin neat plates and ISO 14125 for composite parts. Moreover, ILSS tests were carried out at room temperature for composite parts according to ASTM D2344.

#### 2.2.6. Density

The densities of the developed resin and composite plate were determined in accordance with the liquid displacement method (ASTM D792-20).

#### 2.2.7. Fibre and Void Volume Fraction

The burn-off method described in ASTM D3171-22 was used to determine the fibre volume fraction, Vf, and the void volume fraction, Vv, of the composite samples.

## 3. Results and Discussion

### 3.1. Rheological Characterisation

**Figure 1:**
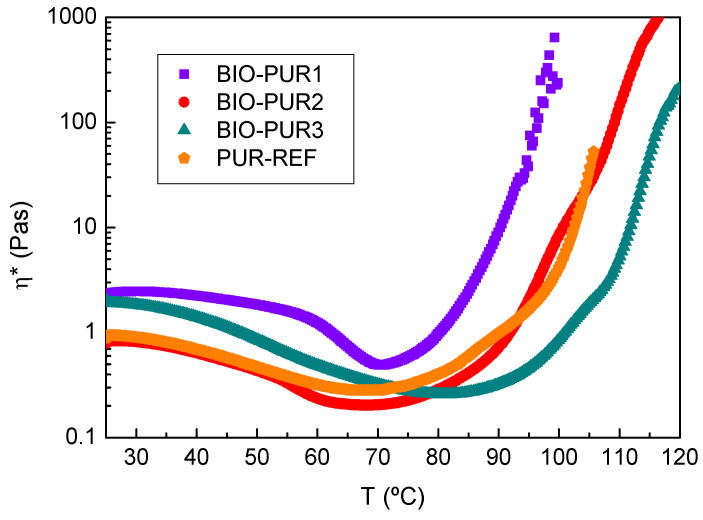
Complex viscosity evolution with temperature for PUR systems.

Viscosity results from oscillatory temperature sweep tests are shown in Figure 1. Temperature sweep rheology tests show the high reactivity of BIO-PUR1 (Figure 1). The viscosity decreased with increasing temperature until the curing started, accompanied by an abrupt increase in the viscosity at 70 °C. Therefore, this system would start to react in a few seconds at the target process temperatures and would not have the necessary latency.

In the case of BIO-PUR2 and BIO-PUR3, the results showed an improvement in both the reactivity control and initial viscosity, which was attributed to the glycerol incorporation to the formulation. This crosslinking agent presents very low viscosity at room temperature, causing a decrease in the initial viscosity of BIO-PUR2 and BIO-PUR3. On the other hand, the presence of the secondary and lower reactivity hydroxyl groups of the glycerol delays the BIO-PUR curing reaction. The reactivity and viscosity evolution of BIO-PUR2 were comparable with those of the PUR-REF petrochemical-origin catalysed system. The addition of the catalyst to the BIO-PUR further decreased the reactivity, as can be seen in the BIO-PUR3 viscosity evolution curve. In this case, the viscosity increase starts at 85 °C making the system suitable for a broader range of RTM process temperatures.

### 3.2. Differential Scanning Calorimetry

**Figure 2:**
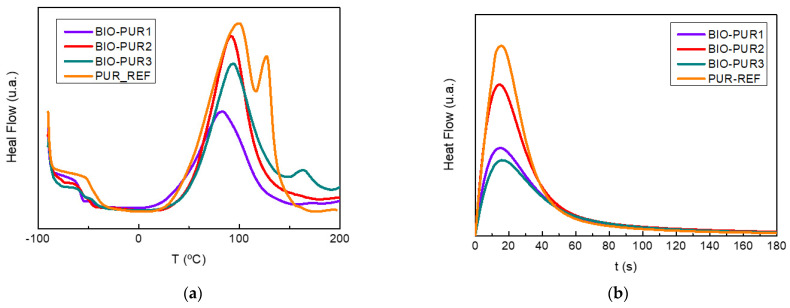
(**a**) Dynamic DSC thermograms and (**b**) isothermal DSC thermograms for PUR systems.

The curing reaction of BIO-PUR1, BIO-PUR2, BIO-PUR3, and PUR-REF was also characterised by both dynamical and isothermal DSC tests. Figure 2a shows the thermograms obtained at 10 °C min^−1^. As can be seen, the reaction is delayed for the BIO-PUR2 and BIO-PUR3 systems, supporting the results obtained in the rheology tests. The peak of the BIO-PUR1 system shows a maximum at 83 ℃, whereas it appears at 91 and 94 °C for BIO-PUR2 and BIO-PUR3, respectively. Moreover, in the case of BIO-PUR3 and the reference system, the shape of the heat flow curve changes, and a second peak can be seen at higher temperatures. This is attributed to the two-step catalytic mechanism [45].

Another critical issue in the case of ultra-fast curing resins is the heat released during the curing process. The resins can have temperature instabilities due to the fast heat production speed, especially in the case of thick laminates, as in the case of the target application, hindering their processability. The total heat of reaction, taken as the value obtained at 10 °C min^−1^, was 301 J g^−1^ for the PUR-REF system, whereas for the biobased systems BIO-PUR1, BIO-PUR2, and BIO-PUR3, the values were 121, 205, and 175 J g^−1^, respectively.

The total heat of reaction of BIO-PURs increased with the total hydroxyl or isocyanate group content (Table 1). This is the reason why BIO-PUR2 and BIO-PUR3 presented higher total heat of reaction than BIO-PUR1. Moreover, other factors, such as the effect of incorporating the delayed action catalyst to the isocyanate, should also be considered. BIO-PUR3 presented lower heat of reaction than the uncatalysed system BIO-PUR2. This is related to the urethane prepolymer formation during the catalyst component’s preparation. In this step, some isocyanate groups react with hydroxyl groups, and the heat released in this step was not measured in the later DSC curing analysis [45].

Figure 2b shows the results obtained in the isothermal tests at 120 °C (target process temperature). In the case of BIO-PUR1, a maximum degree of cure of 0.99-1 (full cure) was obtained, whereas for PUR-BIO2 and PUR-BIO3, values of 0.97 and 0.96 were calculated, respectively. The reference petrochemical origin system, PUR-REF, had a maximum degree of cure of 0.94. In all cases, the maximum degree of cure obtained is considered good enough to avoid post-curing.

### 3.3. Dynamical Mechanical Analysis

**Figure 3:**
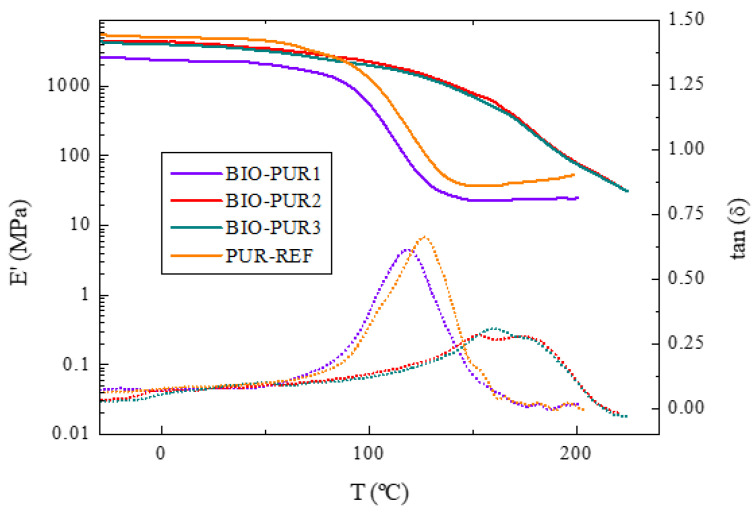
Storage modulus (E’, continuous line) and loss factor (tan δ, dashed line) vs temperature for PUR systems.

Figure 3 shows the DMA results (storage modulus and tan δ) as a function of temperature for the PUR systems. The T_g_ of each material was taken as the temperature value of the maximum of tan δ (Table 2). The T_g_ of BIO-PUR1 was 119 ℃, which is too tight for automotive composite parts, where a minimum of 120 ℃ is targeted. The reference system, PUR-REF, had a T_g_ of 124 ℃. On the other hand, for BIO-PUR2 and BIO-PUR3, higher T_g_ values were obtained, i.e., 161 and 167 ℃, respectively.

**Figure 4:**
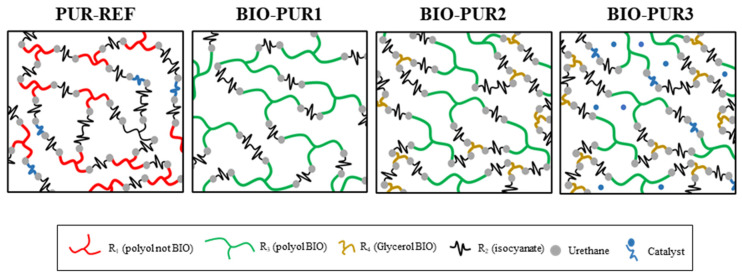
Scheme of PUR/BIO-PUR system three-dimensional network.

The changes in T_g_ are attributed to the low molecular weight and high functionality of the BIO-Gly that produces an increase in the crosslinking density (Figure 4). Moreover, the storage modulus was also affected by the decrease on the network mobility, significantly increasing for BIO-PUR2 and BIO-PUR3 systems compared with BIO-PUR1.

### 3.4. Mechanical Properties

Table 3 summarises the mechanical properties of the PUR systems. It can be observed that the addition of BIO-Gly resulted in an increase in the flexural modulus and strength. The results of the modulus are in accordance with those obtained in the DMA tests (Table 2). Moreover, BIO-PUR2 and BIO-PUR3 maintained the flexural strain compared with BIO-PUR1. As it can be seen, when the glycerol was incorporated, as in BIO-PUR2 and BIO-PUR3, the mechanical properties were comparable with the properties of the petrochemical origin reference system, showing their suitability for structural applications. It was also observed that the addition of the catalyst did not produce any significant difference on the mechanical properties.

### 3.5. Modelling and Process Simulation

**Figure 5:**
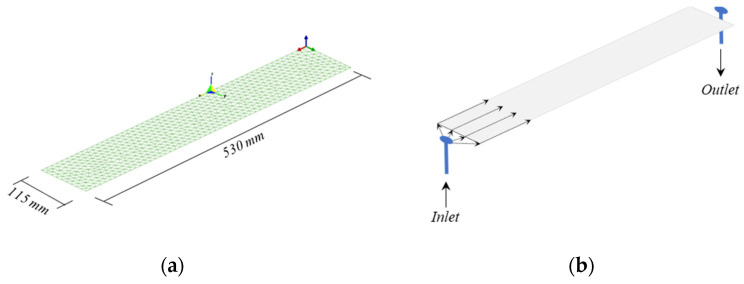
(**a**) Mesh and (**b**) injection strategy used in the simulation.

The next step was to evaluate the suitability of the developed systems (BIO-PUR2 and BIO-PUR3) for the target application. For this, the RTM process of a leaf spring reinforced with 47% volume content of high-fatigue-resistance (ultra-fatigue) unidirectional glass fibre was simulated. These parts are usually produced with a linear, lateral injection strategy, with the resin inlet at the middle point of the part and the outlets at the ends (Figure 5), so for the evaluations, only half-length of the real part was considered.

The flow of the polyurethane resin through the glass fibre fabric can be described by Darcy’s law (Equation (3)).

(3) $$Q=-\frac{S\;K\;}{ɸ\;\eta }\nabla P$$

where *Q* denotes the resin flow rate, *K* is the preform permeability, *S* is the cross-sectional area, ɸ is the porosity, *η* is the resin viscosity, and *P* represents the pressure. For the preform, a nominal permeability of 1.35 × 10^−10^ m^2^ was considered for this fibre volume content [47].

On the other hand, as previously mentioned, resin viscosity depends on resin curing temperature and time. Therefore, the curing reaction and viscosity evolution were modelled to obtain the rheo-kinetic equations.

For the cure kinetic modelling, the degree of cure obtained from the dynamic and isothermal DSC tests was fitted using the Kamal–Sourour model (Equation (4)) [48]. In order to consider the diffusion effect and have a good fitting in all the degree of cure ranges, the model was completed with a diffusion factor *F*(*α*) (Equation (5)) [49].

(4) $$\frac{d\alpha }{dt}=\left(k_{1}e^{-\frac{E1}{T}}+k_{2}e^{-\frac{E2}{T}}\alpha^{m}\right){\left(1-\alpha \right)}^{n}F\left(\alpha \right)$$

(5) $$F\left(\alpha \right)=\frac{1}{1+e^{\left(Ed\left(\alpha -\alpha_{c}\right)\right)}}$$

where

(6) $$E_{d}=E_{d1}+E_{d2}\;T$$

and

(7) $$a_{c}=a_{c1}+a_{c2}\;T$$

are temperature-dependent adjustable parameters (Equations (6) and (7)).

In this equation, *α* is the degree of cure, dα/dt is the reaction rate, n and m are the reaction orders, and T is the temperature. The variables *k*_1_, *E*_1_ and *k*_2_, *E*_2_ are the pre-exponential factors and activation energies of the *n*th- and *m*th-order reactions, respectively, and *F*(*α*) corresponds to the diffusion factor.

**Figure 6:**
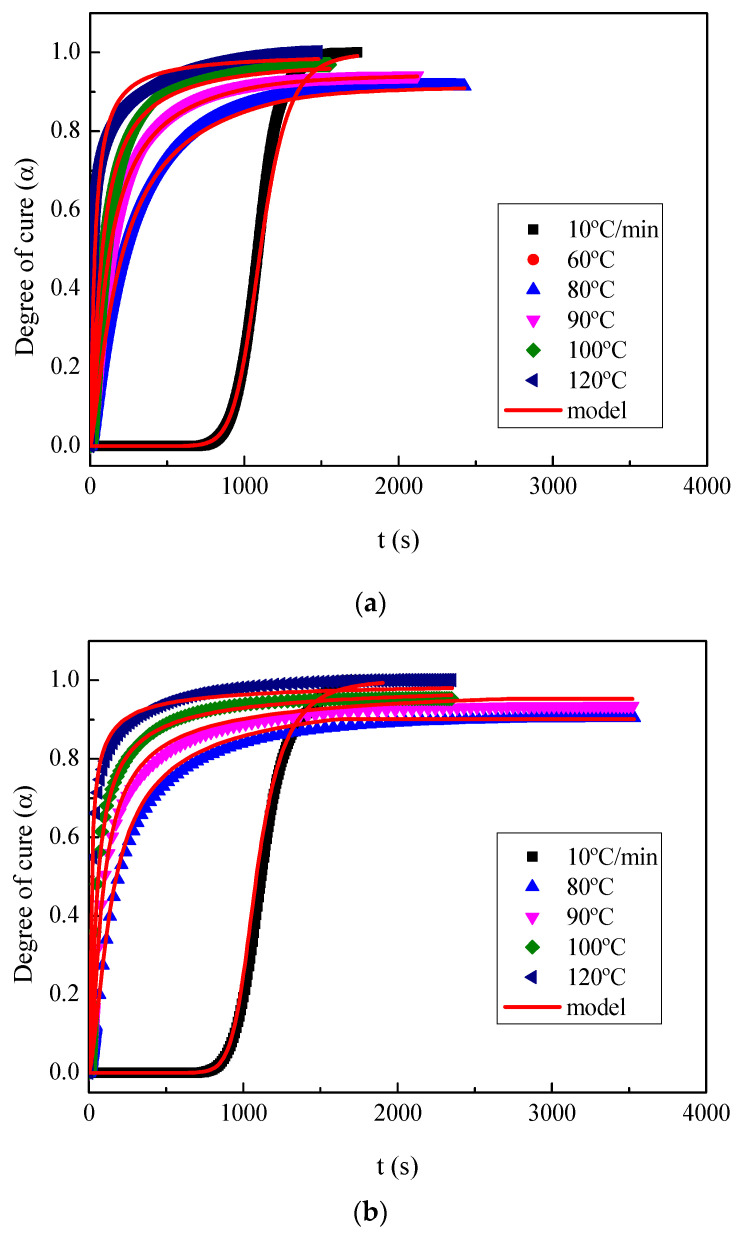
Degree of cure curves from DSC isothermal and dynamic tests (symbols) and model fitting (red lines) for (**a**) BIO-PUR2 and (**b**) BIOPUR3.

The kinetic model parameters for each of the BIO-PUR2 and BIO-PUR3 systems are summarised in Table S1. As it can be seen in Figure 6, there is a good correlation between the experimental results and proposed models.

**Figure 7:**
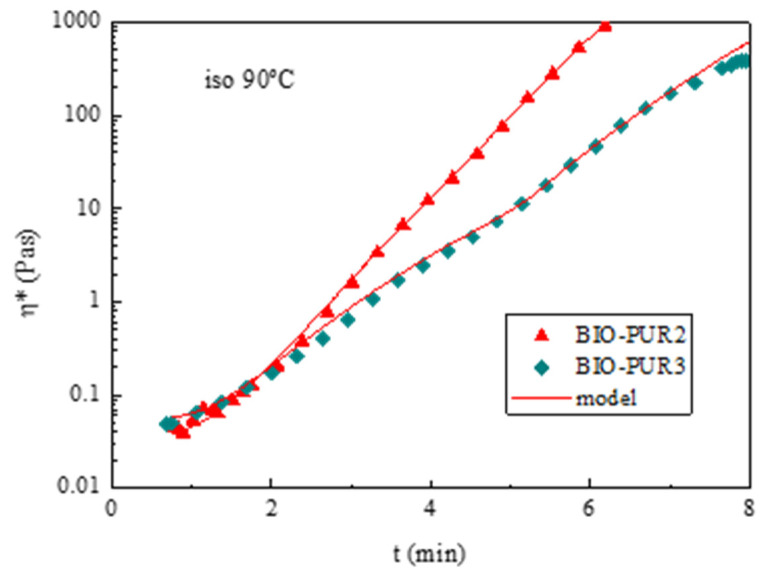
Viscosity evolution with time at 90 °C for BIO-PUR2 and BIO-PUR3. Experimental results (symbols) and model (red lines).

For viscosity modelling, the results obtained from the time and temperature sweep tests for BIO-PUR2 and BIO-PUR3 were fitted to the following equation based on the Castro–Macosko model (Equation (8)) [50]:

(8) $$\eta =\eta_{0}e^{\frac{E}{T}}{\frac{1}{\left(1-\alpha \right)}}^{p1+p2\alpha }$$

where *η* is the resin viscosity at a given degree of cure (*α*), temperature (*T*), and activation energy (*E*); and *η*_0_, *p*1, and *p*2 are adjustable parameters. The viscosity model parameters for BIO-PUR2 and BIO-PUR3 are summarised in Table S2. As it can be seen in Figure 7, there is a good agreement between the experimental results and model for both systems.

The temperature used for the simulations was 120 °C, which is a standard temperature used to produce composite automotive parts. In addition, two different injection strategies were considered, namely constant pressure and constant flow.

**Figure 8:**
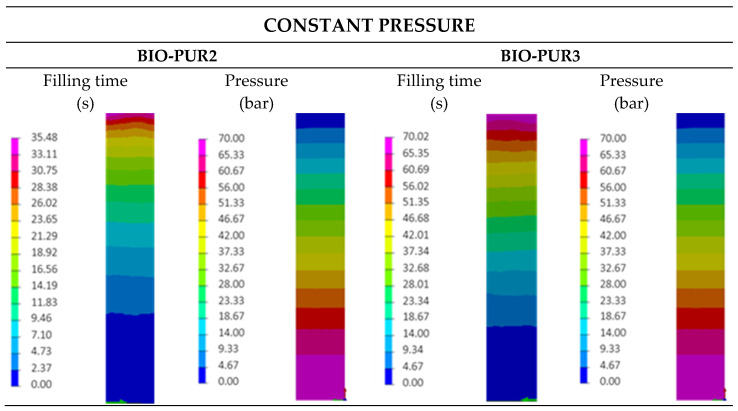

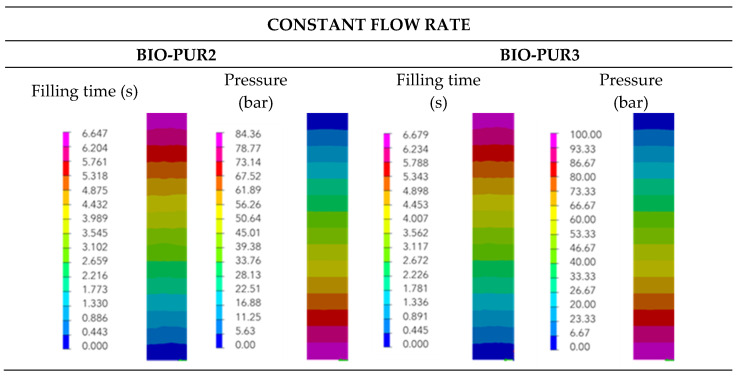
Leaf spring RTM process simulation.

In the case of constant pressure injections, the simulations were carried out at 70 bar. BIO-PUR2 system simulation results were satisfactory (Figure 8), showing the processability of BIO-PUR2 at moderate pressures. At pressures higher than 70 bar, this system could fill the mould in just a few seconds. After the mould filling, the curing was completed in eight minutes without the need of additional post-curing. BIO-PUR2 provided the good combination of latency and fast curing to be suitable for RTM. The BIO-PUR3 system had a longer filling time due to its higher initial viscosity, but showed a latent behaviour, achieving a curing degree and viscosity of 0.34 and 612 mPa s, respectively, after the filling. It was also observed that compared with BIO-PUR 2, BIO-PUR3 required three additional minutes to reach full curing.

### 3.6. Composites Manufacturing, Testing, and Validation

In order to further validate the BIO-PUR formulation suitability, composite plates were manufactured and tested. The reinforcement and fibre content used was the same as in the target application process simulations (47% fibre volume content of high-fatigue-resistance (ultra-fatigue) unidirectional glass fibre), but the process conditions had to be changed due to a 3-bar injection pressure limit of the used glass cover mould. The glass cover mould allowed to compare the real process and the RTM simulations and validate the models previously used. In addition, in this case, the less reactive BIO-PUR3 system was selected. Moreover, the selected process temperature was also lower than the target 120 °C. In this case, the filling was performed at 60 °C and at pressures ranging from 0.5 to 3 bar, and then it was post-cured for 1 h at 120 °C.

**Figure 9:**
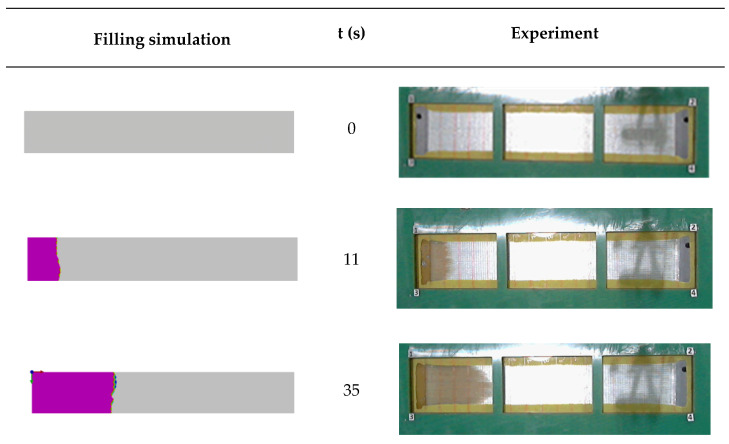

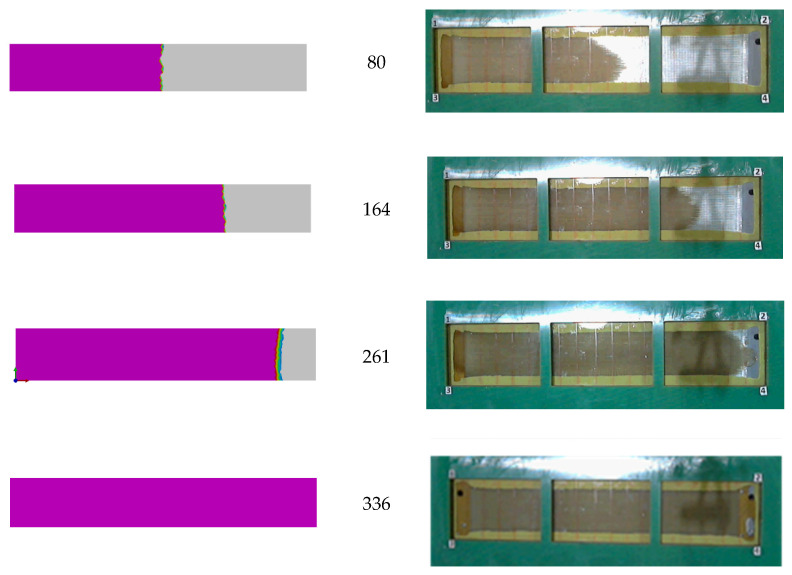
Comparison between simulation and real RTM process with BIO-PUR3 formulation.

Figure 9 shows the comparison between the experimental and simulation results. As can be seen, although there is a good agreement and the model fitting could be considered good enough, the real process is slightly quicker than the theoretical one (Figure S2). This is probably due to the presence of higher permeability zones that modified the flow pattern, as can be seen in Figure 9.

Once manufactured, characterisation tests were carried out to test the composite plate quality. The results are shown in Table 4. The BIO-PUR3 composite exhibits excellent mechanical properties with a modulus and flexural strength of 35 GPa and 1000 MPa, respectively. The ILSS value of the coupons, extracted at different plate lengths, showed a very small deviation showing that constant quality was attained. The void content of the coupons was less than 1.25% in all the cases. The fibre volume content was also constant and in accordance with the theoretical one (47%). Moreover, the T_g_ value is also higher than 120 ℃, fulfilling the automotive structural parts requirements.

## 4. Conclusions

The addition of a biobased glycerol, BIO-Gly, to a high functionality and high hydroxyl value castor-oil-based polyol was proven to be efficient for increasing the crosslinking density, T_g_, and mechanical properties of a structural BIO-PUR.

Moreover, the system showed a viscosity profile similar to that of the reference petrochemical origin structural PUR system, with the required latency and reactivity for the RTM process. The addition of a delayed action catalyst allowed to further delay the curing if necessary.

The suitability of the resin system was demonstrated through the simulation and manufacturing of high-fibre-volume unidirectional glass fibre composites. The quality of the obtained composites was good, with less than 2% of voids and a T_g_ value higher than 120 ℃. Regarding the mechanical properties, a flexural modulus higher than 35 GPa and strength higher than 1000 MPa were obtained, fulfilling the requirements for automotive structural applications.

## Figures and Tables

**Table 1:** Composition of PUR systems studied.

System	Components Ratio (Pbw)						
	Part A			Part B			Renewable Content (%)
	Polyol	Glycerol	BDDE	Isocyanate	LiCl	DAS	
PUR-REF	100	-	7	181	3	12	4
BIO-PUR1	100	-	-	117	-	-	37
BIO-PUR2	100	22	-	233	-	-	29
BIO-PUR3	100	22	7	267	2	9	27

**Table 2:** Storage modulus at room temperature and T_g_ values for BIO-PUR/PUR systems.

System	T_g_	E’ (25 °C)
	°C	GPa
PUR-REF	124	3.4
BIO-PUR1	119	2.3
BIO-PUR2	161	3.2
BIO-PUR3	167	3.0

**Table 3:** Flexural properties of PUR systems.

Systems	Flexural Strength	Flexural Modulus	Flexural Strain
	MPa	GPa	%
PUR-REF	139.0 ± 1.6	3.3 ± 0.1	6.6 ± 0.2
BIO-PUR1	92.8 ± 4.3	2.2 ± 0.1	6.6 ± 0.7
BIO-PUR2	124.2 ± 2.3	2.9 ± 0.1	6.9 ± 0.3
BIO-PUR3	127.6 ± 0.9	3.0 ± 0.1	6.5 ± 0.1

**Table 4:** Final properties of BIO-PUR-based composite.

BIO-PUR3		
Method	Properties	Value
Mechanical properties	Flexural strength (MPa)	1009 ± 61
	Flexural modulus (GPa)	36.8 ± 1.0
	Flexural strain (%)	2.8 ± 0.2
	ILLS (MPa)	65 ± 2
DMA	T_g_ (℃)	138
Liquid displacement method	Matrix density at 25 ℃ (ρ_m_) (g cm^−3^)	1.21
Burn-off method	Void content (Vv) (%)	−0.28 ± 0.96
	Fibre volume content (Vf) (%)	48.2 ± 0.9

## Data Availability

Not applicable.

## Supplementary Materials

The following supporting information can be downloaded at: https://www.mdpi.com/article/10.3390/polym14214553/s1, Table S1: Kinetic model parameters for BIO-PUR systems, Table S2: Viscosity model parameters for BIO-PUR systems, Figure S1: Polyurethane resin system preparation, Figure S2: Flow front evolution for BIO-PUR3. Experimental (symbols) and simulation (?) results.
