# Supplementary material for: Development of a Novel Biobased Polyurethane Resin System for Structural Composites
Source: Polymers (Basel). 2022 Oct 27;14(21):4553. doi: 10.3390/polym14214553 (PMC9654683; doi:10.3390/polym14214553)
Supplement: Supplementary file 1 [file polymers-14-04553-s001.zip › polymers-1954092-supplementary.pdf]

Article

Development of a Novel Biobased Polyurethane Resin System for Structural Composites

Oihane Echeverria-Altuna [1], Olatz Ollo [1], Izaskun Larraza [2], Cristina Elizetxea [1], Isabel Harismendy [1,*] and Arantxa Eceiza [2,*]

[1] TECNALIA, Basque Research and Technology Alliance, Science and Technology Park, Gipuzkoa Mikeletegi Pasealekua 2, 2009 Donostia-San Sebastian, Spain
[2] 'Materials + Technologies' Research Group (GMT), Department of Chemical and Environmental Engineering, Faculty of Engineering of Gipuzkoa, University of the Basque Country, Plaza Europa 1, 20018 Donostia-San Sebastian, Spain
* Correspondence: isabel.harismendy@tecnalia.com (I.H.); arantxa.eceiza@ehu.eus (A.E.)

Supplementary Materials

Table S1. Kinetic model parameters for the BIO-PUR systems.

		BIO-PUR2	BIO-PUR3
K_1	s^{-1}	2.59E+09	2.04E+09
E_1	ºK	5.76E+04	1.01E+04
K_2	s^{-1}	7.32E+05	7.57E+04
E_2	ºK	6.55E+03	5.56E+03
m		2.42E-01	4.96E-01
n		2.02E+00	2.39E+00
α_{c1}		-3.08E-01	-9.71E-01
α_{c2}		3.41E-03	5.00E-03
E_{d1}		-1.71E+02	7.28E+02
E_{d2}		8.10E-01	2.77E+01

Table S2. Viscosity model parameters for the BIO-PUR systems.

		BIO-PUR2	BIO-PUR3
η_0	Pa s	1.73E-07	2.93E-06
E	ºK	7011	6300.2
p1		-2.2	-4.4
p2		10.8	10.0

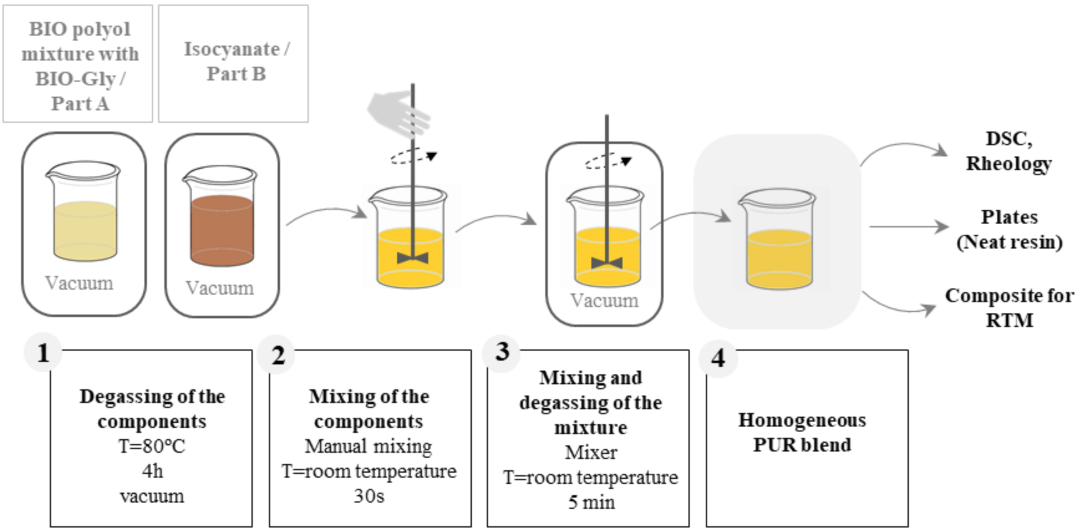

Figure S1. Polyurethane resin system preparation.

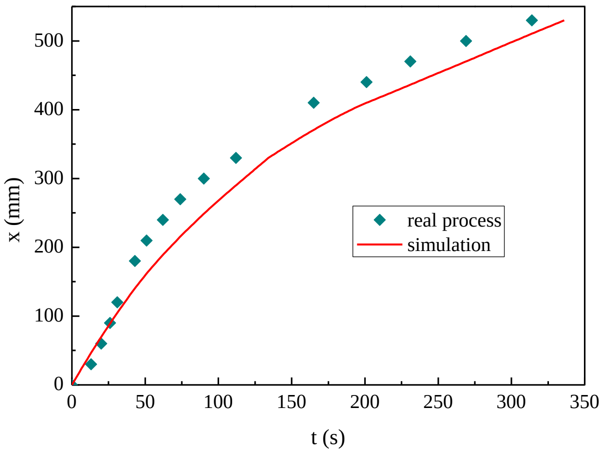

Figure S2. Flow front evolution for BIO-PUR3. Experimental (symbols) and simulation (—) results.
